# Contributions of a Rehabilitation Nursing Program in the Self-Care of Women Undergoing Breast Surgery

**DOI:** 10.3390/nursrep13020080

**Published:** 2023-06-15

**Authors:** Tânia Rodrigues, Maria Teresa Moreira, Andreia Lima, Rita Fernandes, Bárbara Gomes

**Affiliations:** 1 Research Center for Health Technologies and Services (CINTESIS@RISE), Santa Maria Health School, 4049-024 Porto, Portugal; rita.fernandes@santamariasaude.pt; 2 Research Center for Health Technologies and Services (CINTESIS@RISE), Institute of Research, Innovation and Development Fernando Pessoa Foundation, 4200-253 Porto, Portugal; tmoreira@ufp.edu.pt (M.T.M.);; 3 Faculty of Medicine, University of Porto (FMUP), 4200-319 Porto, Portugal; 4 Nursing School of Porto, 4200-072 Porto, Portugal; bgomes.28021955@gmail.com

**Keywords:** breast cancer, rehabilitation nursing, rehabilitation program, axillary lymph node dissection, mammectomy

## Abstract

Background: Although surgical treatments for breast cancer have exhibited advanced interventions, axillary lymph node dissection can limit functionality and compromise women’s self-care. This study aims to assess the effectiveness of a rehabilitation nursing program in improving self-care performance in women undergoing breast surgery with axillary lymph node dissection. Methods: This quantitative, quasi-experimental study involved 48 women recruited from a main hospital between 2018 and 2019. The participants completed a three-month rehabilitation program at home. The evaluation instrument used was the DASH questionnaire. This study was not registered. Results: The functionality of the upper limb ipsilateral to the surgery improved significantly (*p* < 0.001) after the implementation of the program, influencing the participants’ capacity for self-care, including washing/drying their hair, washing their back, and wearing a shirt. The average DASH total score increased from 54.4 to 8.1 after the program. Conclusions: The rehabilitation nursing program positively influenced the participants’ self-care ability. Incorporating rehabilitation nursing programs into breast cancer treatment can improve self-care performance and the overall quality of patients’ lives. This study was not registered.

## 1. Introduction

Breast cancer is the most commonly diagnosed cancer in women worldwide, with the exception of East Africa, and is the leading cause of cancer-related death in 11 regions worldwide [1]. The probability of developing breast cancer during a woman’s life is 1 in 8, and the likelihood of dying from it is 1 in 33. In Portugal, breast cancer accounts for approximately 30% of all cancer cases in women [2].

The possibility of breast mutilation, which is often seen as a symbol of femininity, can generate a range of emotions, such as insecurity, uncertainty regarding the chances of a cure, and fear of death, amputation, treatment, body image changes, disease progression, or loss of work or a partner [3,4]. Women with breast cancer experience a range of concerns, including ambiguity, insecurity, the anticipation of losses, and vulnerabilities [5]. Female breasts are not only glandular structures; they are also associated with femininity, sexuality, motherhood, and attractiveness. As a result, breast cancer and its treatment can significantly affect a woman’s physical health and her quality of life, sexuality, and body image [6].

Contrary to the past, a breast cancer diagnosis does not necessarily mean death. In the last two decades, there have been significant advances in innovative surgical techniques, radiation approaches, and drugs, making it a curable disease [7]. Breast cancer treatment strategies vary depending on the stage of cancer, including the size, affected site, invasion of other organs, and the patient’s physical condition. Treatment options include targeted therapies, hormone treatment, radiotherapy, and surgery [8]. Some treatments are local, such as surgery and radiotherapy, while others are systemic, including chemotherapy, immunotherapy, and targeted therapy. However, any treatment may cause side effects [9].

Surgery may be indicated to remove the tumor, screen for axillary lymph node invasion, or alleviate symptoms in advanced-stage disease.

Cancer patients often report changes in their Activities of Daily Living (ADLs), which are essential for independent living. About one-third of adults with this condition experience difficulty, and half of women need assistance to perform basic and instrumental ADLs, such as personal hygiene, walking, transfers, housework, shopping, and transportation. Personal hygiene, walking, and transfers are the most important areas affected [10].

After breast cancer treatment, many symptoms occur due to decreased patient functionality, limiting their ability to carry out ADLs [11]. Treatment’s negative effects incapacitate most women, leading to isolation, absence from work, and difficulties with domestic activities, including taking care of children and participating in religious practices [12].

Surgery can have negative impacts on the physical and emotional well-being of breast cancer patients, including anxiety, menopausal symptoms, body weakness, restricted range of motion, frozen shoulder, muscle weakness, poor body image, lymphedema, fatigue, and pain, which can limit patients’ ability to perform self-care and other daily activities [12,13,14]. The function of the upper limb is particularly important for self-care in women who have undergone breast surgery. Additionally, if associated with Axillary Lymph Node Dissection (ALND), it increases the risk of developing changes in the arm and shoulder, decreased range of motion and muscle strength, pain, lymphedema, and difficulties carrying out ADL. Upper extremity lymphedema is a chronic and common complication caused by damage to lymph nodes and lymphatic vessels during ALND. The incidence of postoperative lymphedema in breast cancer patients has been reported to be 15–33%. Lymphedema often happens from half a year after ALND to even several years afterward. Lymphedema and its related symptoms seriously affect the quality of patients’ lives. The severity of adverse reactions in the breast and in the upper limb consequently decreases the patients’ ability to perform self-care. Additionally, if, on the one hand, the progress achieved in terms of prevention, treatment, and recovery of breast cancer have contributed to the increase in survivors, on the other hand, they have created new challenges, both for health professionals and for these women, regarding rehabilitation and quality of life.

Breast cancer surgery can cause sequelae in women in the long term as a result of the treatment, such as anatomical changes, functional changes (a decrease in muscle strength, aerobic capacity and mobility of the upper limb), chronic pain syndrome, phantom breast syndrome, axillary web syndrome, lymphedema and other pain syndromes: rotator cuff, arthralgias, adhesive capsulitis, cervical radiculopathy, and brachial plexopathy [15]. All these alterations directly or indirectly cause changes in women’s daily lives [11,13,14] and changes in their QoL [11,13,16,17] in several domains, with repercussions for QoL [15].

A woman undergoing breast surgery with ALND has a higher risk of developing alterations in the upper limb. Rehabilitation is the best option to prevent complications, recover shoulder range of motion and prevent shoulder, arm and hand disability [18], maintain functional capacity, and decrease the risk of losing important skills or independence [19]. Evidence indicates that rehabilitation is beneficial in these women’s recovery, mostly after 3 months [20,21,22]. Failure to carry out rehabilitation leads to limitations in the participation of ADLs. The movement of the upper limb is qualitatively less fluid (during the reaching function), even if it apparently performs the reaching movement correctly [23]. Therefore, it is particularly important to study the effect of nursing intervention on controlling the occurrence of postoperative lymphedema. A holistic approach by health professionals throughout this process (from diagnosis to rehabilitation) is essential, enabling women to experience their health and well-being process. Rehabilitation is important for cancer patients, but few receive rehabilitation interventions due to a lack of investment in this area [24]. Rehabilitation programs are not widely available, and resources are limited. A nurse who is a specialist in rehabilitation nursing has knowledge and is in a prominent position to promote self-care, prevent complications and train women with breast cancer to perform exercises that allow them to recover, maintain or improve their functional capacity and enhance their independence. However, in Portugal, its intervention is still not very visible. To address this issue and investigate the benefits of a rehabilitation program for women who have undergone breast surgery with ALND, the following research question arises: What is the relationship between the implementation of a rehabilitation program and the self-care performance capacity in women undergoing breast surgery with ALND?

## 2. Materials and Methods

A quantitative, quasi-experimental study was conducted on a non-probabilistic convenience sample of 48 women who have undergone breast surgery with ALND. The study was conducted after the patients were discharged from the nursing consultation at the Breast Center of a Portuguese Hospital Center between February 2018 and June 2019. The inclusion criteria for the study were healed surgical wounds, age over 18, patients with decision-making capacity, without osteoarticular limitations before surgery, and those without a rehabilitation program. The sample size calculation was performed using G * Power 3.1.9.2 [25], resorting to the *t*-test as a reference, namely the *t*-test for paired samples with an effect size of 0.5, an α error of probability of 0.05, and (1-β error prob) = 0.95.

The participants were recruited at their homes after prior contact with one researcher. Data collection was carried out before and three months after the implementation of the rehabilitation nursing program, following a favorable opinion from the institution’s Ethics Committee (Opinion 298/17). Participation in the program was voluntary, and all participants signed free and clear informed consent. The procedures respected the ethical principles inscribed in the Declaration of Helsinki.

The rehabilitation nursing program was designed based on available scientific evidence. The rehabilitation nursing program was built in several stages. Thus, initially, a bibliographical analysis was performed on several sources of information (database, repositories, and aggregator systems) and based on the collected evidence, the rehabilitation nursing program was created, later being reviewed and validated by a group of experts in the field.

It consisted of an individual teaching session on lymphedema risk reduction measures, mobilization exercises for the upper limb ipsilateral to the surgery and the cervical spine (a flyer with photographs of the exercises was provided), and an indication of scar and upper limb massages. After a group of experts had legitimized the program, the leading researcher implemented it at the participants’ homes. At the participants’ homes, the main researcher explained the program, which included teaching, instructing and training the set of exercises (mobilization exercises for the upper limb ipsilateral to the surgery and the cervical spine) and scar and arm massage, with flyers detailing measures to reduce the risk of lymphedema and photographs of the exercises being provided as support. Regarding the resources for carrying out the exercises, none in particular were needed, and materials used in everyday life were used, namely a broom handle and a bath towel. As for the scar and arm massage, the moisturizing cream that the women applied on a daily basis was used. For three months, the women performed the rehabilitation nursing program daily, lasting 45 min and repeating each exercise ten times [20,21,22,26]. The principal investigator supervised the program at the participants’ homes once a week. The principal investigator visited the participants’ homes to supervise the program, promote their adherence, and detect complications. In addition, the fact that the program was at home prevented frequent trips to the hospital; promoted more comfort, as patients were in their usual environment; and there was also no economic impact. A data collection instrument was used to measure the variables, including a sociodemographic characterization section, a clinical characterization, and the questionnaire “Disabilities of the Arm, Shoulder and Hand (DASH)”, which was applied before and after the rehabilitation nursing program.

DASH was developed by the American Academy of Orthopedic Surgeons, together with the Institute for Work and Health in Toronto, Canada [27], to measure physical disability and symptoms of the upper limbs in people with disorders of the upper limbs (hand, wrist, elbow, and shoulder) [28], and permission via email from the authors. It is a self-administered questionnaire that includes physical, psychological, and social functioning [29], consisting of 30 questions, providing a DASH function/symptoms score [DASH-FS]. The score is a summation of responses on a scale of 1–5, resulting in a final value ranging from zero (no disability) to 100 (severe disability). It also comprises two groups of optional questions made up of 4 items, called modules related to work and modules related to sport/music, which are scored similarly. The questions assess the degree of difficulty in performing a variety of physical activities because of arm, shoulder, or hand problems [21 items], investigate pain intensity, pain-related activity, tingling, weakness, stiffness [5 items], and the effects of upper limb problems on social activities, work, sleep, and self-image [4 items]. All items have five response options, ranging from “no difficulty or no symptoms” to “unable to perform activities or extreme symptoms” [30]. The minimum detectable change is 8–15 points, indicating a significant impairment in upper limb function at a score of 15 or higher. Scores between 16 and 40 suggest a problem with upper limb function that is still tolerable, while scores above 40 indicate that patients are unable to work [31]. The tool demonstrates good internal consistency/cross-sectional reliability (Cronbach’s α = 0.92–0.98) and test–retest reliability (intraclass correlation coefficient 0.93–0.98) [31].

The collected data were analyzed and presented using Statistical Package for the Social Sciences—IBM SPSS^®^ Statistics, version 25.0 for Windows, resorting to specific methods and techniques.

To identify associations between sociodemographic variables and self-care performance capacity with the rehabilitation program, adherence and homogeneity tests were conducted for all quantitative variables in the study, and Fisher’s coefficient was used. The Wilcoxon test was used to compare subscale values before and after the implementation of the rehabilitation nursing program. Statistical tests considered a confidence interval of 95% and a significance level of *p* < 0.05.

## 3. Results

The study sample consists of 48 women who underwent breast surgery with ALND, who met the inclusion criteria. The results obtained show that the distribution of ages is positively asymmetric (Fisher’s asymmetry coefficient is 0.45), which means that younger and moderate ages predominate. The minimum age was 28 years. Predominate ages range between 40 and 50, where there is a sharp drop in the number of women, registering gradual decreases in the number of women in the following ages, with a maximum of 79 years. The mean age is 49.2 years, almost coincident with the median, which is 49 years (i.e., half of women are aged up to 49 years), the first quartile is 42 years (i.e., a quarter of women are aged up to 42 years) and the third quartile is 57 years old (that is, three quarters of women are aged up to 57 years old), which shows that ages are concentrated in low and moderate values. As a result, the dispersion is reduced, reflected in the value of the coefficient of variation (23.2%). The participants’ qualifications ranged from none (one) to the post-doctoral degree (one). Other qualifications whose scores stood out were the 9th grade (22.9%), the bachelor’s degree (18.8%), the 12th grade (16.7%), the 6th grade (14.6%), and the 4th grade (8.3%).

With regard to the marital status variable, we found that Married women were the majority (27 women or 56.3%), followed by Single (9 women or 18.8%), Divorced (7 women or 14.6%), de facto union (3 women or 6.3%) and Widowed women (2 women or 4.2%).

Among the participants, there were 29 different occupations, of which 18.8% were unemployed, 10.4% were seamstresses, 8.3% were housewives, 8.3% were pensioners, and 4.2% were operational assistants.

With regard to the age at which the first child was born, we found that the distribution of ages is strongly asymmetrically positive (the asymmetry coefficient of Fisher is 1.1), which means that younger to moderate ages predominate, between 18 years old (the minimum age) and 28 years old, where there is a big drop in number of women. From that age onwards, the number of women is reduced, with a maximum age of 43 years. Thus, the average age is 25.7 years, almost coinciding with the median, which is 25 years (that is, half of women had their first child by age 25 years), the first quartile is 21 years (i.e., a quarter of women had their first child by at age 21) and the third quartile is 28 years (i.e., three-quarters of women had their first child up to age 28), which shows that ages are concentrated in low values. In as a result, the dispersion is reduced, which is reflected in the value of the coefficient of variation (23.9%).

As for the number of children, we found that two children stand out (21 women or 43.8%), followed by one child (15 women or 31.3%), no children (6 women or 12.5%), three children (5 women or 10.4%) and four children (1 woman or 2.1%). As a result, the lowest numbers strongly predominate; that is, from 0 (the minimum number) to 2 children, with few women having three or four children (the maximum number).Thus, by analyzing the results described, the average number of children is 1.6, the median number is two children (that is, half of women have up to two children), the first quartile is one child (i.e., a quarter of women have up to one child) and the third quartile is two children (i.e., three quarters of women have up to two children). There are large differences in the number of children among women, reflected in a high coefficient of variation (58%).

Regarding the type of breast surgery with ALND, the most frequent was the modified radical mastectomy (47.9%), followed by the tumorectomy (37.5%), the total mastectomy (12.5%), and the radical mastectomy (2.1%). As for family history of breast cancer, we found that only a minority have a family history (19 women or 39.6%), whereas the majority do not (29 women or 60.4%).

Supplementary Table S1 presents the scores from the DASH questionnaire before and after the intervention program (scores range from 0 to 100) and compares the scores by item.

Additionally, the scores were compared using the Wilcoxon test for paired samples, since the same women were assessed at both moments. The *p*-value of the test is also presented in Supplementary Table S1, with a significance level of 5%. Except for the item related to sexual activities, it was observed that the degree of disability decreased after the program in all other items (with an extremely low *p*-value), indicating improvement. However, for the item of sexual activities, no significant difference in the degree of disability was found between the two moments.

Regarding self-care abilities, specifically “wash or blow dry your hair” (item 13), “wash your back” (item 14), and “put on a jumper/a pullover sweater” (item 15), significant improvements were observed after the program, with a *p*-value under 0.001 for all three items. Before the program, 29.2% of the women reported inability and 29.2% reported difficulty washing and blow drying their hair. However, after the program, 97.9% reported no difficulty in doing so. Similarly, before the program, 56.3% of the women reported an inability to wash their backs, but after the program, 77.1% reported no difficulty in doing so. Before the program, 37.5% of the women reported difficulty putting on jumper/a pullover sweater, and 31.3% reported some difficulty in doing so. Nevertheless, 93% stated no difficulty putting on jumper/a pullover sweater after the program.

Regarding the relationship between the rehabilitation nursing program and symptom improvement, significant improvements were observed for pain, numbness, weakness, and stiffness in the arm, shoulder, and hand (corresponding to questions DASH 24, 26, 27, and 28, respectively) after the program. Before the program, 50% of the women reported pain, numbness (29.2% some and 39.6% a lot), weakness (41.7% some and 41.7% a lot), stiffness (31.3% some and 50% a lot). After the program, these percentages decreased to 70.8%, 60.4%, 70.8%, and 89.6%, respectively, with all symptoms showing significant improvement with a *p*-value under 0.001.

Table 1 shows the characterization of the DASH questionnaire before and after the intervention program, comparing the scores in the two moments using the Wilcoxon test for paired samples (since they refer to the same women in two different moments). The *p*-value of the test is also indicated in Table 1 (5% significance level).

Before the program, the DASH scores ranged from 40 to 80, with a predominant range of 50 to 70 indicating moderate to severe disability levels. The mean score was 54.4, slightly lower than the median of 57.5. The first quartile was 44.4, and the third quartile was 67.7, indicating a moderate to severe disability level among most women. However, some low scores resulted in a moderate dispersion, as perceived in the coefficient of variation value of 34.7%. In summary, women with moderate to severe levels of disability predominated before the program.

After the program, scores below four were predominant, with a sharp drop in the number of women with higher scores. There were few women with scores above 15, and only one had a score higher than 30. This score was equal to 32.5, the maximum (much lower than the maximum in the scale). The mean score was 8.1, slightly higher than the median of 5. The first quartile was 1.7, and the third quartile was 10.8, indicating a very low level of disability among most women. The concentration of very low scores and some slightly higher scores resulted in a large dispersion, as reflected in the coefficient of variation value of 103.3%. In summary, women with a very low level of disability strongly prevailed after the program.

The comparison between before and after the program showed a significant reduction in scores, shifting from a predominant range of moderate to severe disability levels before the program to a strong predominance of very low levels after it. The Wilcoxon test statistic was 2252.5, with a *p*-value of less than 0.001, indicating a clear and significant improvement in disability levels after the program. Table 1 above clearly depicts this difference. Therefore, it was concluded that, on average, the degree of disability of women decreased after the program.

## 4. Discussion

The rehabilitation program aimed to improve the ability of women undergoing breast surgery with ALND to perform self-care using the upper limb ipsilateral to the surgery, as well as to educate and empower them regarding scar and upper limb care in relation to the risk of lymphedema.

Prior to the program, we observed that women with a moderate to a severe degree of disability prevailed, with half of them scoring up to 57.5, indicating high disability (DASH > 50.7). These results contrast with those of previous studies [32,33], which found that disability was mostly mild or absent in women with ALND.

After the program, we found that the mean score was 8.1, with half of the women scoring up to 5, indicating a predominance of very low disability. This is a significant improvement compared to reference values for low disability (DASH < 43.5), showing a drastic decrease in disability after the intervention. When comparing the degree of disability before and after the intervention program, we found that the degree of disability decreased significantly after the program. Our results agree with a study [21] that found that rehabilitation exercises for the upper limbs initiated early after mastectomy with ALND significantly improved shoulder, arm, and hand disability over three months postoperatively.

The rehabilitation program aimed to improve the ability of women undergoing breast surgery with axillary lymph node dissection to perform self-care using the upper limb ipsilateral to the surgery, as well as to educate them on scar and upper limb care to prevent lymphedema. The study results showed that before the program, women predominantly had a moderate to severe degree of disability in the arm, shoulder, and hand, with half of the women presenting a score of up to 57.5, indicating a high level of disability. Nonetheless, after the intervention, the level of disability significantly decreased, with half of the women presenting a score of up to five, indicating a very low degree of disability.

The study found that physical and functional limitations in the upper limb ipsilateral to the surgery affected the women’s ability to perform activities of daily living (ADLs), such as washing and blow drying their hair, washing their backs, and putting on a jumper/a pullover sweater. These results are consistent with previous studies that have reported changes in functional capacity and ADL limitations in women undergoing breast surgery [34,35,36].

Regarding the relationship between the rehabilitation nursing program and symptom improvement, the study found that pain, numbness, weakness, and stiffness in the arm, shoulder, and hand significantly improved after the program. These findings are consistent with previous studies that have reported the effectiveness of rehabilitation in improving pain [23,35,37,38] and arm symptoms [37].

Our findings suggest that the rehabilitation nursing program developed and implemented was as effective as an early rehabilitation program. In similar programs, it is recommended that the teaching and training of women be carried out by nurses specialized in rehabilitation nursing, using follow-up consultations, and home visits, supplemented with the provision of written material and images.

The study presents some limitations that should be taken into account. One of those is the small size of the sample for statistical associations, which is attributed to difficulties in participant recruitment. Furthermore, more than the short follow-up period of three months may be required to assess the long-term durability of the program’s effects. In addition, the study relied on self-reported outcomes, which may be subject to individual interpretation and perception. Another limitation of this study is related to the fact that the researcher goes to the participants’ homes. On the one hand, this promotes adherence; on the other hand, it can lead to a “Hawthorne effect” or other threats to “External Validity”.

It also did not use objective measures, such as range of motion or muscle strength testing, which could provide more precise and reliable data on the effectiveness of the rehabilitation nursing program. Conducting a pre-surgery assessment (pre-rehabilitation) to identify existing functional levels and deficiencies could contribute to a change in the intervention program and improve postoperative results while reducing hospitalization time. It is suggested that the rehabilitation nurse supervise and accompany the recovery process and intercurrences of these women and that, in the future, specialized teams be created to accompany them, from the moment of the surgical decision through the recovery and follow-up consultation after the end of the program. Nurses specialized in rehabilitation nursing already exist in legislation and in Portuguese hospitals and community institutions of free public service, which can be directed to this area of intervention. We also suggest that more studies be carried out to investigate the benefits of rehabilitation nursing programs on the quality of life of these women, using the most recent evaluation instruments specific to this pathology.

## 5. Conclusions

Breast cancer remains a global health concern despite treatment and early detection advances. Breast cancer survivors often have several physical, cognitive, emotional, psychosocial, family, body image, sexual, and functional impairments in the ipsilateral upper limb, which can interfere with their ability to perform self-care activities and, consequently, their quality of life, which is why it is essential to adopt strategies that promote achievement and maintain the best possible functioning in all dimensions of quality of life. Specialist nurses trained in rehabilitation nursing had expertise in identifying and managing functional changes associated with breast cancer treatment and in the design and implementation of rehabilitation programs to improve upper limb motor function and activities of daily living.

This study demonstrated the effectiveness of a 12-week home-based rehabilitation nursing program for women undergoing breast surgery with axillary lymph node dissection. The program led to significant improvements in the functional capacity of the upper limb on the side of surgery, resulting in an enhanced ability to perform self-care tasks, such as washing and blow drying their hair, washing their backs, and putting on a jumper/pullover sweater. Our findings suggest that investing in rehabilitation programs for women undergoing breast surgery is crucial for optimal functional recovery.

The rehabilitation nursing program effectively improved the functional capacity of the ipsilateral upper limb, as evidenced by the increased scores on the questionnaire and gains in health-related quality of life of women undergoing breast surgery with ALND. This highlights the essential role of specialist nurses in preventing and managing post-surgical complications associated with breast cancer treatment.

## Figures and Tables

**Table 1:** Comparison of DASH scores before and after the intervention program.

Coefficients	Before	After	*p*
DASH			<0.001
Minimum	5.0	0.0	
Maximum	86.7	32.5	
Average	54.4	8.1	
1st quartile	44.4	1.7	
Median	57.5	5.0	
3rd quartile	67.7	10.8	
Coef. Asymmetry	−0.57	1.2	
Standard deviation	18.9	8.4	
Coefficient of variation	34.7%	103.3%	

## Data Availability

Not applicable.

## Supplementary Materials

The following supporting information can be downloaded at: https://www.mdpi.com/article/10.3390/nursrep13020080/s1, Table S1: Characterization of the DASH questionnaire before and after the intervention program and item comparison.

## Public Involvement Statement

There was no public involvement in any aspect of this research.

## References

[1] Ferlay J., Colombet M., Soerjomataram I., Mathers C., Parkin D., Pineros M., Znaor A., Bray F. (2019). Estimating the global cancer incidence and mortality in 2018: GLOBOCAN sources and methods. Int. J. Cancer.

[2] National Cancer Registry Breast Cancer Is the Most Common Cancer in Women and Represents about 30% of All Cases of Cancer in Women. https://ron.min-saude.pt/pt/tumor/top5/mama/epidemiology/.

[3] Silva G.F., Bastos K.D., Araujo A.J., Bishop T.C., Oliveira G.A., Schulz R.S. (2018). Women undergoing mastectomy: Sentimental and emotional aspects. Rev. Enferm. Contemp..

[4] Vicente Pardo J.M., López-Guillén García A. (2017). Problems and psychological factors in the return to work after prolonged temporal incapacity due to breast cancer. Med. Segur. Trab..

[5] Ghahari S., Fallah R., Behnam L., Rad M.M., Farrokhi N., Ghayoomi R. (2018). Concerns and worries in women with breast cancer: A qualitative study. J. Pharm. Res. Int..

[6] Słowik A.J., Jabłoński M.J., Michałowska-Kaczmarczyk A.M., Jach R. (2017). Evaluation of quality of life in women with breast cancer, with particular emphasis on sexual satisfaction, future perspectives and body image, depending on the method of surgery. Psychiatr. Pol..

[7] Ng Z.X., Ong M.S., Jegadeesan T., Deng S., Yap C.T. (2017). Breast cancer: Exploring the facts and holistic needs during and beyond treatment. Healthcare.

[8] Akram M., Iqbal M., Daniyal M., Khan A.U. (2017). Awareness and current knowledge of breast cancer. Biol. Res..

[9] Ghaemi S.Z., Keshavarz Z., Tahmasebi S., Akrami M., Heydari S.T. (2019). Conflicts women with breast cancer face with: A qualitative study. J. Family. Med. Prim. Care.

[10] Neo J., Fettes L., Gao W., Higginson I.J., Maddocks M. (2017). Disability in activities of daily living among adults with cancer: A systematic review and meta-analysis. Cancer Treat. Rev..

[11] Light K.M., Heins M., Verloop J., Ezendam N.P., Smorenburg C.H., Korevaar J.C., Siesling S. (2019). The impact of health symptoms on health-related quality of life in early-stage breast cancer survivors. Breast Cancer Res. Treat..

[12] Iddrisu M., Aziato L., Dedey F. (2020). Psychological and physical effects of breast cancer diagnosis and treatment on young Ghanaian women: A qualitative study. BMC Psychiatry.

[13] Mokhatri-Hesari P., Montazeri A. (2020). Health-related quality of life in breast cancer patients: Review of reviews from 2008 to 2018. Health Qual. Life Outcomes.

[14] Wilson D.J. (2017). Exercise for the patient after breast cancer surgery. Semin. Oncol. Nurs..

[15] Lovelace D.L., McDaniel L.R., Golden D. (2019). Long-term effects of breast cancer surgery, treatment, and survivor care. J. Midwifery Women’s Health.

[16] Zabit F., Iyigun G. (2019). A comparison of physical characteristics, functions and quality of life between breast cancer survivor women who had a mastectomy and healthy women. J. Back Musculoskelet. Rehabil..

[17] Hsiao F.H., Kuo W.H., Jow G.M., Wang M.Y., Chang K.J., Lai Y.M., Chen Y.T., Huang C.S. (2019). The changes of quality of life and their correlations with psychosocial factors following surgery among women with breast cancer from the post-surgery to post-treatment survivorship. Breast.

[18] Chan K.S., Zeng D., Leung J.H., Ooi B.S., Kong K.T., Yeo Y.H., Goo J.T., Chia C.L. (2020). Measuring upper limb function and patient reported outcomes after major breast cancer surgery: A pilot study in an Asian cohort. BMC Surg..

[19] Amatya B., Khan F., Galea M.P. (2017). Optimizing post-acute care in breast cancer survivors: A rehabilitation perspective. J. Multidiscip. Healthc..

[20] Akezaki Y., Nakata E., Kikuuchi M., Tominaga R., Kurokawa H., Okamoto M., Hamada M., Aogi K., Ohsumi S., Sugihara S. (2021). Investigation of factors affecting early quality of life of patients after breast cancer surgery. Healthcare.

[21] Kikuuchi M., Akezaki Y., Nakata E., Yamashita N., Tominaga R., Kurokawa H., Hamada M., Aogi K., Ohsumi S., Tsuji T. (2021). Risk factors of impairment of shoulder function after axillary dissection for breast cancer. Support Care Cancer.

[22] Paolucci T., Bernetti A., Paoloni M., Capobianco S.V., Bai A.V., Lai C., Pierro L., Rotundi M., Damiani C., Santilli V. (2019). Therapeutic alliance in a single versus group rehabilitative setting after breast cancer surgery: Psychological profile and performance rehabilitation. Biores Open Access.

[23] Paolucci T., Capobianco S.V., Bai A.V., Bonifacino A., Agostini F., Bernetti A., Paoloni M., Cruciani A., Santilli V., Padua L. (2020). The reaching movement in breast cancer survivors: Attention to the principles of rehabilitation. J. Bodyw. Mov. Therap..

[24] Alfano C.M., Pergolotti M. (2018). Next-generation cancer rehabilitation: A giant step forward for patient care. Rehabil. Nurs. J..

[25] Faul F., Erdfelder E., Lang A.-G., Buchner A. (2007). G* Power 3: A flexible statistical power analysis program for the social, behavioral, and biomedical sciences. Behav. Res. Methods.

[26] ACSM (2023). American College of Sports Medicine, Leading the Way. Indianapolis: American College of Sports Medicine. https://www.acsm.org/docs/default-source/files-for-resource-library/cancer-infographic-sept-2022.pdf..

[27] Knaut L.A., Moser A.D., Melo S.D., Richards R.R. (2010). Translation and cross-cultural adaptation of the American Shoulder and Elbow Surgeons Standardized Shoulder Assessment Form (ASES) to Portuguese for assessing shoulder function. Rev. Bras. Reumatol..

[28] Hudak P.L., Amadio P.C., Bombardier C., Beaton D., Cole D., Davis A., Hawker G., Katz J.N., Makela M., Marx R.G. (1996). Development of an upper extremity outcome measure: The DASH (disabilities of the arm, shoulder, and head). Am. J. Ind. Med..

[29] Davies C.C., Lengerich A., Bugajski A., Brockopp D. (2018). Detecting change in activity using the Patient-Specific Functional Scale with breast cancer survivors. Rehabil. Oncol..

[30] Ruivo R.M., Pezarat-Correia P., Carita A.I. (2015). Portuguese version of the American Shoulder and Elbow Surgeons Standardized Shoulder Assessment Form (ASES). RPOT.

[31] Angst F., Pap G., Mannion A.F., Herren D.B., Aeschlimann A., Schwyzer H.K., Simmen B.R. (2004). Comprehensive assessment of clinical outcome and quality of life after total shoulder arthroplasty: Usefulness and validity of subjective outcome measures. Arthritis Care Res..

[32] Riaz S., Soomro S., Manzoor S., Kumar D., Mohammad D. (2015). Assessment of disability of arm, shoulder and hand using DASH scoring system after axillary dissection for breast cancer. Pak. J. Surg..

[33] Marazzi F., Masiello V., Marchesano D., Boldrini L., Luzi S., Ferrara P.E., Amabile E., Piccari D., Landi F., Moschella F. (2019). Shoulder girdle impairment in breast cancer survivors: The role of range of motion as predictive factor for dose distribution and clinical outcome. Tumori J..

[34] Lang A.E., Kim S.Y., Dickerson C.R., Milosavljevic S. (2020). Measurement of objective shoulder function following breast cancer surgery: A scoping review. Phys. Ther. Rev..

[35] Jare N.S., Malawade M. (2021). Effect of graded theraband exercise on myofascial dysfunctions in breast cancer survivors. Indian J. Forensic. Med. Toxicol..

[36] Venâncio A.P., Gardenghi G. Role of Physiotherapy and Its Benefits in Post-Mastectomy Surgery. https://ceafi.edu.br/site/wp-content/uploads/2019/05/atuacao-da-fisioterapia-e-seus-beneficios-no-pos-operatorios-de-mastectomia-1.pdf.

[37] Cho Y., Do J., Jung S., Kwon O., Jeon J.Y. (2016). Effects of a physical therapy program combined with manual lymphatic drainage on shoulder function, quality of life, lymphedema incidence, and pain in breast cancer patients with axillary web syndrome following axillary dissection. Support Care Cancer.

[38] Alpozgen A.Z., Ozdincler A.R., Karanlik H., Agaoglu F.Y., Narin A. (2017). Effectiveness of pilates-based exercises on upper extremity disorders related to breast cancer treatment. Eur. J. Cancer Care.

