# Supplementary material for: Contributions of a Rehabilitation Nursing Program in the Self-Care of Women Undergoing Breast Surgery
Source: Nurs Rep. 2023 Jun 15;13(2):913–22. doi: 10.3390/nursrep13020080 (PMC10304504; doi:10.3390/nursrep13020080)
Supplement: Supplementary file 1 [file nursrep-13-00080-s001.zip › nursrep-2348651-supplementary.pdf]

Table S1. Characterization of the DASH questionnaire before and after the intervention program and item comparison.

	Before the program						After the program						
**DASH**	**1***	**two***	**3***	**4***	**5***	**6***	**1***	**two***	**3***	**4***	**5***	**6***	
**Item**	**%**	**%**	**%**	**%**	**%**	**%**	**%**	**%**	**%**	**%**	**%**	**%**	***P***
1	14.6	8.3	12.5	22.9	41.7	0.0	60.4	12.5	10.4	10.4	6.3	0.0	<0.001
2	83.3	2.1	10.4	2.1	0.0	2.1	100.0	0.0	0.0	0.0	0.0	0.0	0.006
3	58.3	6.3	16.7	4.2	14.6	0.0	93.8	2.1	4.2	0.0	0.0	0.0	<0.001
4	47.9	6.3	27.1	14.6	4.2	0.0	93.8	4.2	2.1	0.0	0.0	0.0	<0.001
5	20.8	12.5	29.2	27.1	10.4	0.0	75.0	16.7	6.3	2.1	0.0	0.0	<0.001
6	2.1	2.1	20.8	12.5	62.5	0.0	81.3	14.6	2.1	0.0	2.1	0.0	<0.001
7	6.3	6.3	20.8	18.8	47.9	0.0	72.9	14.6	8.3	2.1	0.0	2.1	<0.001
8	20.8	8.3	20.8	12.5	33.3	4.2	91.7	2.1	2.1	2.1	0.0	2.1	<0.001
9	25.0	6.3	20.8	25.0	22.9	0.0	89.6	8.3	2.1	0.0	0.0	0.0	<0.001
10	12.5	4.2	20.8	20.8	41.7	0.0	79.2	10.4	8.3	0.0	2.1	0.0	<0.001
11	8.3	2.1	4.2	12.5	72.9	0.0	41.7	8.3	12.5	12.5	18.8	6.3	<0.001
12	2.1	2.1	12.5	10.4	72.9	0.0	87.5	10.4	0.0	2.1	0.0	0.0	<0.001
13	18.8	4.2	18.8	29.2	29.2	0.0	97.9	2.1	0.0	0.0	0.0	0.0	<0.001
14	4.2	10.4	10.4	18.8	56.3	0.0	77.1	20.8	2.1	0.0	0.0	0.0	<0.001
15	6.3	10.4	31.3	37.5	14.6	0.0	93.8	6.3	0.0	0.0	0.0	0.0	<0.001
16	62.5	4.2	12.5	10.4	10.4	0.0	97.9	0.0	2.1	0.0	0.0	0.0	<0.001
17	87.5	2.1	6.3	2.1	2.1	0.0	100.0	0.0	0.0	0.0	0.0	0.0	0.012
18	8.3	6.3	25.0	25.0	35.4	0.0	83.3	14.6	0.0	0.0	2.1	0.0	<0.001
19	8.3	6.3	20.8	29.2	35.4	0.0	87.5	6.3	4.2	0.0	2.1	0.0	<0.001
20	39.6	6.3	31.3	8.3	12.5	2.1	95.8	4.2	0.0	0.0	0.0	0.0	<0.001
21	43.8	0.0	16.7	6.3	18.8	14.6	56.3	2.1	12.5	4.2	25.0	0.0	0.786
22	33.3	10.4	14.6	35.4	6.3	0.0	93.8	6.3	0.0	0.0	0.0	0.0	<0.001
23	2.1	6.3	10.4	50.0	31.3	0.0	87.5	8.3	4.2	0.0	0.0	0.0	<0.001
24	10.4	4.2	50.0	33.3	2.1	0.0	70.8	22.9	6.3	0.0	0.0	0.0	<0.001
25	6.3	4.2	50.0	39.6	0.0	0.0	62.5	31.3	6.3	0.0	0.0	0.0	<0.001
26	22.9	4.2	29.2	39.6	4.2	0.0	60.4	25.0	10.4	4.2	0.0	0.0	<0.001
27	12.5	4.2	41.7	41.7	0.0	0.0	70.8	20.8	8.3	0.0	0.0	0.0	<0.001
28	8.3	8.3	31.3	50.0	2.1	0.0	89.6	8.3	2.1	0.0	0.0	0.0	<0.001

29	31.3	0.0	41.7	22.9	4.2	0.0	91.7	6.3	2.1	0.0	0.0	0.0	<0.001
30	12.5	6.3	8.3	56.3	16.7	0.0	64.6	4.2	4.2	27.1	0.0	0.0	<0.001

Legend: Scale: 1* - No difficulty; 2* - Mild difficulty; 3* - Moderate difficulty; 4* - Severe difficulty; 5* - Unable; 6* - Did not answer.
